# Prospective adherence to specific immunotherapy in Europe (PASTE) survey protocol

**DOI:** 10.1186/s13601-015-0060-0

**Published:** 2015-04-27

**Authors:** Melina Makatsori, Gianenrico Senna, Constantinos Pitsios, Ramon Lleonart, Ludger Klimek, Carlos Nunes, Maia Rukhadze, Barbara Rogala, Radoslaw Gawlik, Petr Panzner, Oliver Pfaar, Moises Calderon

**Affiliations:** Royal Brompton and Harefield NHS Trust, London, UK; Imperial College London, National Heart and Lung Institute, London, UK; Allergy Department, Azienda Universitaria-Ospedaliera Integrata, Verona, Italy; Private Allergy Clinic, Athens, Greece; Allergy Department, University Hospital of Bellvitge, Barcelona, Spain; Center for Rhinology and Allergology, Wiesbaden, Germany; Centro de Imunoalergologia do Algarve, Portimao, Portugal; Allergy & Immunology Centre, Tbilisi, Georgia; Medical University of Silesia, Allergology & Clinical Immunology, Katowice, Poland; Faculty of Medicine in Pilsen, Charles University Prague, Immunology and Allergology, Pilsen, Czech Republic; Department of Otorhinolaryngology, Head and Neck Surgery, Universitätsmedizin Mannheim, Medical Faculty Mannheim, Heidelberg University, Mannheim, Germany

**Keywords:** Adherence, Sublingual immunotherapy, Subcutaneous immunotherapy, Hymenoptera venom allergy, Respiratory allergic disorders, Online survey, Online register

## Abstract

**Background:**

Adherence to allergen immunotherapy is important for its effectiveness. There is currently limited data available on allergen immunotherapy adherence outside of clinical trials i.e. in real-life clinical practice. As part of a European Academy of Allergy and Clinical Immunology Immunotherapy Interest group initiative, we endeavoured to design a survey in order to prospectively evaluate adherence to subcutaneous and sublingual immunotherapy across different European countries.

**Method/Design:**

The inclusion criteria for this prospective, multi-country survey were set as: adults, starting clinically indicated allergen immunotherapy for respiratory allergic disorders or Hymenoptera venom allergy. An online survey was designed in order to enrol participants and assess adherence to immunotherapy.

Eight countries (Czech Republic, Georgia, Germany, Greece, Italy, Poland, Portugal, Spain) were selected to reflect different parts of Europe and differences in allergens and routes of immunotherapy administration. Each country has an allocated National co-ordinator that has identified local Allergy departments willing to enrol participants in this survey.

Each participant will be followed up for a total of three years. In order to assess adherence, a 4-monthly follow-up form detailing any missed doses and reasons will be completed online. In case of a participant discontinuing treatment, reasons for this will be recorded.

**Discussion:**

The use of online survey software has enabled us to make this survey a reality and reach clinicians in different countries. Forty-five centres have enrolled a total of over 1,350 participants. It is hoped that this prospective real life survey will enable us to gain a better understanding of reasons that affect adherence to subcutaneous and sublingual immunotherapy and assist in developing ways to improve this.

## Introduction

The lack of adherence to medication is an emerging and worldwide problem in the treatment of chronic diseases. It is responsible for a decrease of treatment efficacy, increased hospitalisations, morbidity and mortality, and is a significant burden for patients as well as for society and healthcare systems [1]. Rates of treatment discontinuation are reported to be between 20-40% for acute illnesses, increasing to 30-60% for chronic conditions and reaching 80% in preventative interventions [2]. In the United States alone, costs due to lack of adherence have reached the figure of 100 billion dollars mainly due to unjustified hospitalisations [3]. Accordingly, medical research has been showing growing interest in this area. On PubMed, the number of studies concerning this subject has more than doubled within the last ten years, increasing from 1,349 in 2003 to 3,376 in 2013.

Adherence to treatment is the complex result of different determinants related to the patient, the disease, the therapy, the patient/physician relationship and the healthcare system [1,2,4]. All of these factors interact in different ways in a single individual. Furthermore, the complexity of the treatment schedule as well as the route of administration can negatively affect adherence [5,6].

Allergic rhinitis is a very common disease with a significant socio-economic burden, due to direct (medications and doctor visits) and indirect costs (loss of productivity/working days) [7]. Currently, the worldwide prevalence of allergic rhinitis in adults ranges from 10-30%, but in children it reaches 37% in Western countries [7]. Discontinuation of pharmacological therapy is a problem shared by allergic rhinitis as well as chronic illnesses [6]. As far as allergen immunotherapy (AIT) is concerned, adherence represents the most critical issue. AIT significantly reduces the clinical symptoms at an early stage as well as the use of concomitant medications, as reported by randomised controlled trials and meta-analyses [7,8]. However, only after a long-term course, whose recommended length is at least three years, it is possible to obtain a long lasting effect following withdrawal and to reduce the risk of development of asthma and/or new sensitisations [9-16]. Immunotherapy for Hymenoptera venom allergy usually lasts between three and five years. Adherence to this is thought to be higher given the life-threatening reactions to venom, however this has not been formally assessed [17].

Rates of adherence for individual patients are usually reported as the percentage of the prescribed doses of the medication actually taken by the patient over a specified period. However, the evaluation of adherence is far from standardised. In fact, many methods have been proposed to measure adherence. Each of them has advantages and disadvantages but none of these can be currently considered the gold standard [1]. Methods for assessing adherence to medication can generally be divided into three categories:

- Subjective measurements based on what is reported by the patient or caregiver. These methods, though inexpensive, are not completely reliable, as patients sometimes can deny the lack of regular medication intake.
- Objective assessment based on the count of pills or the examination of pharmacy refill records. The commonest drawback is that the absent pills are considered actually taken by the patients. The use of electronic devices can be more accurate, but too costly in daily life.
- Measurement of drug levels or drug metabolites in serum or urine. The feasibility of this method is limited to only a few drugs (such as digoxin, anti-epileptics, theophylline, lithium). For asthmatic patients on regular oral steroids the evaluation of serum cortisol can be helpful [6,18].

Besides these technical limitations, a further methodological disadvantage is that if the patients are aware that adherence is an outcome of the study a bias is immediately introduced [1]. For this reason adherence data from randomised controlled trials may not reflect a true representation as participants are strictly followed and observed. More consistent data are therefore likely to be collected from real life studies.

In addition, the wide heterogeneity of immunotherapy studies may account for the conflicting results and the difficulty in drawing definite conclusions. Studies published so far have been carried out in different populations, different countries and according to different designs (retrospective, cross sectional, prospective). Furthermore, patients were treated with various allergen extracts and according to different schedules. Moreover, the methods used to measure adherence were also dissimilar from study to study [19].

Interestingly, although an adherence rate is considered generally acceptable when higher than 80%, a general agreement on this cut off is still lacking [1,19].

Up to now, several studies have been published on subcutaneous immunotherapy (SCIT) [20-34] as well as on sublingual immunotherapy (SLIT) [35-46]. As shown in Tables 1 and 2, the adherence rate is generally low and it varies greatly for both SCIT and SLIT. Comparing the studies according to route of administration, a higher number of SLIT studies reported an adequate (>80%) adherence. On the other hand, SCIT studies included a larger population and were of considerably longer duration.

**Table 1:** **Adherence studies for SCIT**

**Author**	**Study Population (pts)**	**Age (A/C)**	**Study duration**	**Adherence rate (%)**
Chon (1993) [20]	217	A	4 y	50
Lower (1993) [21]	315	C	4 y	56
Tinkelman (1993) [22]	3.349	C,A	1y	65
Ruiz (1997) [23]	247	A	18 m	62
Donahue (1999) [24]	603	C, A	4y	33
Rhodes (1999) [25]	1.033	A	3y	88
More & Hagan (2002) [26]	381	C,A	3y	77
Pajno (2005) [27]	1.886	C	3y	89
Hommers (2006) [28]	296	A	not stated	66
Hankin (2008) [29]	520	C	3y	47 (1 y) 16 (3rd y)
Mahesh (2010) [30]	100	A	not stated	58
Hsu & Reisacher (2012) [31]	139	A	4y	55
Guenechea (2013) [32]	156	A	5y	63
Kiel (2013) [33]	2.796	A	3y	23
Silva (2014) [34]	122	C, A	4y	54

A: adults, C: Children, Y: year, M: month.

**Table 2:** **Adherence studies in SLIT**

**Author**	**Study population (pts)**	**Age (A/C)**	**Study duration**	**Adherence rate (%)**
Marogna (2004) [35]	319	A	3y	85
Lombardi (2004) [36]	86	A	18 m	79-97
Pajno (2005) [27]	806	C	3y	79
Passalacqua (2006) [37]	443	A	6 m	76
Passalacqua (2007) [38]	71	C	6 m	85
Cadario (2008) [39]	40	A	1y	65
Roder (2008) [40]	154	A	2y	77
Chang (2009) [41]	142	A/C	6 m	69
Jansen (2009) [42]	91	A	6 m	95
Ott (2009) [43]	183	A/C	2y	91
Vita (2010) [44]	300	C	2y	30-76
Pajno (2012) [45]	150	C	2y	54
Hsu & Reisacher (2012) [31]	139	A	4y	59
Kiel (2013) [33]	3.690	A	3y	7
Trebuchon (2014) [46]	736	C	2Y	86

A: adults, C: Children, Y: year, M: month.

Many reasons have been reported as being responsible for immunotherapy discontinuation. Among the patient related factors, a younger age and female gender appear to be more frequently related to non adherence [21,25,32,42] whereas severity of the disease seems not to affect the number of dropouts [27-41]. Among the causes for discontinuation, treatment costs are relevant for both subcutaneous and sublingual routes [21,23,26,31,34,37,38,40]. Local side effects following administration may account for SLIT discontinuation in children as well in adults [37,38,40,45], whereas inconvenience due to time consuming schedules, or fear of injections are mainly related to SCIT treatments [21,22,25]. In seasonal allergy, the use of cluster SCIT schedules [20,22] as well as a pre-co-seasonal treatment seem to favour a better adherence [39,40]. The overall analysis of these results does not appear to detect significant differences in adherence according to delivery route [19], but methodological and cultural heterogeneity of the studies currently do not allow for any firm conclusions.

Under the framework of the European Academy of Allergy and Clinical Immunology (EAACI) Immunotherapy Interest Group, we endeavoured to design a survey in order to prospectively evaluate adherence to subcutaneous and sublingual immunotherapy across different European countries. This will include AIT for respiratory allergic disorders as well as Hymenoptera venom allergy.

## Methods

### Objectives

The survey aims to assess adherence to AIT in a real life setting. The primary objectives are to prospectively evaluate the rate of adherence to AIT for inhalant allergies (sublingual and subcutaneous routes) and Hymenoptera venom allergy (subcutaneous) in a three year period in real life across different European countries. The secondary objectives are to identify reasons for lack of adherence and discontinuation of treatment and to explore possible reasons for differences in adherence rates in different countries and different individuals.

### Survey design

This is a prospective, multi-centre, observational survey. The survey will take place in eight countries: Czech Republic, Italy, Germany, Georgia, Greece, Poland, Portugal and Spain. These countries were identified as representative of different areas of Europe and also in order to include a variety of allergens contributing to allergic disease.

This is an online survey that will follow participants for a period of 3 years from the start date of AIT. It was decided to follow participants for a 3-year period as this is the usual length of AIT treatment and there is concern that adherence initially may be relatively good but this may decrease as time passes.

Initially approval for this project was sought from the EAACI Executive Committee. In order to initiate this survey, the two PASTE survey co-ordinators and the EAACI Immunotherapy Interest Group initially contacted the National Allergy Societies of the eight countries to inform them about this survey. Each National Allergy Society identified a "National survey Coordinator” that would be responsible for coordinating all local activities in that country. This involved duties such as identifying study centres willing to participate in the survey and dealing with national regulatory issues such as ethics and any other regulatory requirements.

In order for centres to be able to participate in the survey, they would need to have access to the internet and have an allocated physician in charge who will be in contact with the National survey Coordinator and who will be able to complete the survey questionnaire in English.

### Participant selection and procedure

The survey population consists of adults with IgE mediated respiratory allergies or Hymenoptera venom allergy who will be starting AIT (either SCIT or SLIT) according to accepted clinical standards of practice.

The inclusion criteria were set as: male or female, 18 years of age or over (no upper age limit) and being eligible for immunotherapy to inhalant allergens or Hymenoptera venoms. The exclusion criteria were previous AIT treatment at any time and any contraindications to receiving immunotherapy according to European standards of practice.

Each participating centre coordinator identified any potential participants and assessed whether they met these criteria. In order to enrol a participant, an online enrolment questionnaire was required to be completed by the physician. This form includes information about the participant, details about their allergies and treatment.

For each participant an online follow up adherence form will be completed by the local physician every 4 months. This adherence follow-up form includes questions regarding the number of missed doses, phase of the treatment at the time and any possible reasons for missed doses. The last adherence follow-up form will be completed at 36 months from AIT initiation.

In case of participant withdrawal, a discontinuation online form will be completed. This includes questions including the timing and any identified reasons for stopping AIT.

The enrolment period opened in December 2012 and closed in February 2014.

**Figure 1:**
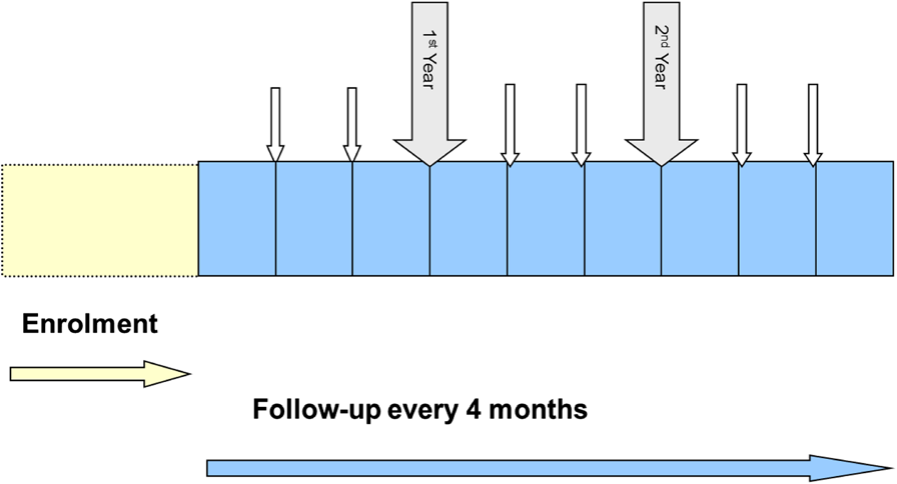
**Study timeline.**

The study timeline is shown in Figure 1.

### Questionnaire design

In order to identify the specific questions and areas to be investigated, the opinion and advice of experts in this field was sought at an EAACI PASTE meeting in Geneva 2012. At that stage, the content of the enrolment form, 4-monthly follow up form and discontinuation form was agreed.

The enrolment includes information about age, sex, education and employment status, allergen sensitisations, condition for which receiving immunotherapy, allergen, route and immunotherapy regime, date of AIT initiation, treatment setting, follow-up arrangements as well as funding for treatment.

For SCIT, recording of any missed doses is likely to be more accurate as participants have to attend the clinic to receive their injection and the physician is always aware of any missed doses or discontinuation. Follow up of SLIT patients varies in different countries and therefore a compromise was reached to enter follow-up data every 4 months as this would fit with the practice in some countries as well as not be a burden to the physicians who have to complete these forms in their own time without any specific funding. Depending on the practice of each country, other data such as pharmacy/prescription records, follow-up appointments and telephone contact will be used to improve patient reporting of any missed doses. It was felt that use of other methods such as treatment diaries may affect results by making patients more aware that they are participating in an adherence survey and thus affecting behaviours.

The follow up adherence form was designed to include questions regarding the number of missed doses in the previous four months, the phase of the treatment at the time i.e. up-dosing or maintenance and any possible reasons for missed doses. Reasons investigated included acute illness, forgetfulness, lack of efficacy, financial costs, time/work commitments, local reaction, systemic reaction, geographical constraints, new medical condition/s, pregnancy and other.

The discontinuation form also explored specific reasons for ending treatment.

The SurveyMonkey® online survey software was selected to be used for the enrolment and follow up data collection. This allows participating centres to enter data from different countries and enable collection in a central database. All data entered is protected by an enhanced security system (Secure Sockets Layer, SSL, a protocol for encrypting information over the Internet) [47].

The follow up survey forms used a skip logic pattern, allowing participating clinicians to avoid certain sections according to their responses in preceding questions.

The survey instruments were tested several times to ensure and improve their functionality and ease of use. Attention was also paid to user interface principles such as colours selected, number of questions per screen, formatting of text and logical structure of the questionnaire.

### Data management

All data collected by the participating centres is entered anonymously in a coded format in order to protect the participants’ identity. A specific country code was allocated to each country. An additional code beginning with the country code was allocated to each centre of that specific country. Furthermore, each participant was given a code incorporating both the country and centre code. This way the enrolment forms can be matched with all the 4-monthly follow up forms completed for each participant at the stage of data analysis.

The data is regularly assessed by the survey co-ordinators in order to check that it is being entered correctly.

### Data protection

In order to further protect the collected data, only the two survey co-ordinators have access to the password protected completed questionnaires. In case of any queries or mistakes made in the data entry, the survey co-ordinators can be contacted to check the entered data and make any necessary changes.

In addition, the SurveyMonkey® facility uses advanced technology for Internet security. To use this tool, as administrator, a unique user name and password must be entered. SurveyMonkey® issues a session “cookie” only to record encrypted authentication information for the duration of a specific session. Once the user accesses secured areas, the Secure Sockets Layer (SSL) technology protects user information using both server authentication and data encryption, ensuring that user data is safe, secure, and available only to authorised persons. SurveyMonkey® is PCI-DSS compliant [47].

### Statistical analysis

#### Sample size estimation

Different proportions of expected drop-out are reported in Table 3. Confidence intervals are computed under this expected proportions assuming 1,000 patients will be recruited.

**Table 3:** **Different proportions of expected drop-out and confidence intervals**

**Patients recruited**	**Adherence (%)**	**Patients adherent to the treatment**	**95% Confidence interval**
1000	95	950	93.5% - 96.3%
1000	90	900	88.0% - 91.8%
1000	85	850	82.6% - 87.2%
1000	80	800	77.4% - 82.4%
1000	75	750	77.7% - 72.2%
1000	70	700	67.1% - 72.8%
1000	65	650	62.0% - 68.0%
1000	60	600	56.9% - 63.1%

#### Data analysis

The primary objective will be summarised as a proportion ± confidence interval 95% (binomial method).

The influence of potential predictors on lack of adherence or discontinuation from the therapy will be explored using a logistic regression. The effect of the hierarchical structure of the data (participants *nested in* centres *nested in* countries) on the results will be eventually explored using multilevel analyses.

### Regulatory and ethical obligations

European Union and specific country ethics and regulatory requirements, including data protection, will be followed. Each National survey co-ordinator will be responsible for addressing the corresponding ethics committee and local regulatory requirements.

### Survey progress

The PASTE co-ordinators are responsible for the proper running of the survey. They are in regular contact with National co-ordinators and individual centres when required. Meetings, conference calls and newsletters are used as a method of updating progress. Following enrolment, the next task is to ensure timely completion of the 4-monthly follow up forms and any discontinuation forms for all the enrolled participants. The data will then go through a further clearing process, followed by data analysis.

## Discussion

Adherence to AIT is an area that requires further investigation as the data currently available is mainly from clinical trials with only a limited number of real life studies and even fewer prospective studies [33,48-50].

This project will provide an insight into this important issue. It will enable an estimation of the rate of adherence to AIT in daily clinical practice for respiratory allergic disorders and Hymenoptera venom allergy. Furthermore, it will allow identification of possible reasons for lack of adherence and discontinuation of treatment. This will provide useful information for changing or modifying clinical behaviours and attitudes towards immunotherapy, in order to improve adherence wherever possible.

The main strengths of this survey are:

1. As a multi-country survey a large number of participants will be recruited and adherence of AIT in a large part of Europe will be assessed. Given the significant number of participants included, the conclusions drawn are more likely to be applicable to a wider population. At the same time, it will enable identification of inter-country differences regarding the use of different routes of administration and reasons for discontinuation.
2. Sharing the same methodology by all participating centres will allow for more reliable and comparable results.
3. In addition to AIT for respiratory allergies, adherence to Hymenoptera venom immunotherapy will also be addressed in this survey. Possible contributing factors leading to lack of adherence or discontinuation will be explored in detail such as extract used, schedule, responsible insect, severity of index reaction and any side effects to AIT.

The use of online survey software has enabled us to make this survey a reality and reach clinicians in 8 different countries. Following the end of the enrolment period, over 1,350 participants have now been enrolled by 45 centres. The main allergens for which participants are having AIT include grass pollens 33.9%, house dust mite 32.3% and 15.3% Hymenoptera venoms. Preliminary one-year follow up data shows that 11.1% of participants have missed on average 2 doses in 4 months whilst less than 2% of participants have discontinued treatment.

Participants will be followed for a total of 3 years. At that point we will be able to analyse all the data and disseminate the findings to the allergy community in order to benefit from this information and depending on the outcomes consider targeting strategies and ways of improving adherence.

## Abbreviations

AIT: Allergen immunotherapy
EAACI: European Academy of Allergy and Clinical Immunology
PASTE: Prospective adherence to specific immunotherapy in Europe
SCIT: Subcutaneous immunotherapy
SLIT: Sublingual immunotherapy
SSL: Secure sockets layer
